# Predictability of the Development of Insulin Resistance Based on the Risk Factors Among Female Medical Students at a Private College in Saudi Arabia

**DOI:** 10.7759/cureus.39112

**Published:** 2023-05-16

**Authors:** Yousria Badawy, Nouf H Aljohani, Gufran A Salem, Fatima M Ashour, Sarah A Own, Nora F Alajrafi

**Affiliations:** 1 Family and Community Medicine, Alexandria University, Alexandria, EGY; 2 Family Medicine, Ibn Sina National College for Medical Studies, Jeddah, SAU; 3 Medicine, Ibn Sina National College for Medical Studies, Jeddah, SAU

**Keywords:** body weight, risk factors, day sleep, daily physical activity, diastolic blood pressure, systolic blood pressure, obesity, life style habits, body mass index, insulin resistance

## Abstract

Background: Insulin resistance (IR) is currently an underlying phenomenon in the etiology of most non-communicable diseases. IR has been proposed as the key linking factor for the metabolic syndrome disease cluster of glucose intolerance.

Objectives: This study's target was to assess the predictability of risk factors for IR among female medical students

Methods: A cross-sectional study involving female medical students was carried out. The sample size was 272, and a suitable non-probability sampling method was employed. A correlation test was done, and a P value less than 0.05 is considered statistically significant. Each participant underwent assessment of anthropometric measurements and biochemical testing. For assessing lifestyle, validated questionnaires on physical activity, sleep pattern, dietary pattern, and stress were adopted. The anthropometric data such as height, weight, and waist circumference were collected. Biochemical testing involved estimation of the postprandial capillary blood glucose level on campus. Additionally, systolic blood pressure and diastolic blood pressure were measured.

Results: The association of lifestyle risk factors with waist circumference as an indicator of IR was done where the majority of those with high waist circumference were physically inactive and more prone to stress which was statistically significant when compared to those with normal waist circumference. However, poor sleep and unhealthy diet were high among those with high waist circumference, but they were not statistically significant.

Conclusion: The correlation of waist circumference as an indicator of IR was highly significant with body mass index, postprandial blood sugar, systolic blood pressure, and diastolic blood pressure. A series of unhealthy lifestyle habits was contributable to developing obesity and therefore IR among medical students in Saudi Arabia.

## Introduction

Background information 

Insulin resistance (IR), a pathological situation characterized by reduced tissue sensitivity to insulin and marked compensatory hyperinsulinemia, has continued to generate interest [1]. For more than a decade, IR has been proposed as the key linking factor for the metabolic syndrome disease cluster of glucose intolerance, hypertension, dyslipidemia, visceral adiposity, and cardiovascular disease [2]. The prevalence of IR and metabolic syndrome is increasing, particularly in developing countries and in younger populations with estimates of prevalence ranging from 20 to 40 percent in different populations [3].

Problem statement

A study was conducted between May 2009 and February 2010 at Sultan Qaboos University, Muscat, Oman. The aim of this study was to assess the prevalence of IR in healthy young Omanis and relate this to their body mass index (BMI). The prevalence of IR in healthy Omani medical students was found to be quite high, and there was a direct correlation between BMI and IR [4]. However, in another study conducted in Mexico City on baseline data from Mexican children and adolescents aged 7-18 years, the result showed IR prevalence of 20.3 % among girls and boys aged 7-18 years [5].

Important factors such as physical activity, diet, sleep, and stress all may contribute to IR, and there are potential interactions between several of these lifestyles [6]. Both observational and interventional studies suggest an important role for physical activity and higher fitness in mitigating IR [7]. Among the physiological systems that respond favorably to physical activity, regular exercise is one of the most demonstrable effects on IR [8].

In addition, overnutrition leads to weight gain and carbohydrate intolerance which lead to the development of IR/hyperinsulinemia, ketogenic diets reduce visceral obesity and increase insulin sensitivity, and the therapeutic carbohydrate-restricted diet can prevent or reverse IR [9]. A cross-sectional study aimed to assess the association of sleep with IR in the 2013/2014 National Health and Nutrition Examination Survey (NHANES). The result revealed that a short and long sleep duration was associated with higher risk of IR and higher scores of metabolic syndrome severity score [10]. Furthermore, stress threatens the homeostasis of adaptive physiological and behavioral changes via the hypothalamic-pituitary-adrenal (HPA) axis. In chronically stressed individuals, dishabituation of the HPA axis is followed by increased secretion of glucocorticoids which influences glucose metabolism resulting in the development of IR [11].

Rational/justification

In recent years, IR has been highlighted as a major health problem throughout the world. Therefore, a study is needed to predict the prevalence as well as risk factors contributing to IR.

## Materials and methods

Study design and setting 

A cross-sectional study was carried out to assess the predictability of the development of IR based on the risk factors among female medical students at a private college, in Saudi Arabia. The studied population consisted of female medical students, attending the medicine program of all academic year levels (from year one to year six). The period during which data were collected was January to December 2022.

The inclusion criterion to be met for participation in this study is to be enrolled in a private medical college.

Exclusion criteria are pregnancy and/or lactation and failure to complete the study measurements.

The sampling technique 

A convenient non-probability sampling technique was used. The sample size determination was based on a single population formula using Epi-info version 7, and the calculated sample size was 214 based on an estimated prevalence of 23%, as established from a previous study in a similar cohort with a margin of error of 5% [12].

Data collection tool 

Each participant underwent an assessment of anthropometric measurements and biochemical testing. A series of questionnaires that assessed demographic data and lifestyle habits related to IR were conducted.

For assessing lifestyle, validated questionnaires on physical activity, sleep pattern, dietary pattern, and stress were adopted. For physical activity, International Physical Activity Questionnaire (IPAQ) - Short Form was used [13]. For sleep, the Single-Item Sleep Quality Scale (SQS) was taken [14]. For dietary pattern, the Healthy Eating Quiz (HEQ) is designed to rate how healthy eating habits were and how they helped to identify areas in the diet that could be improved [15]. For stress, the stress questionnaire designed by the International Stress Management Association (ISMA) was applied [16]. The anthropometric data such as height, weight, and waist circumference were collected. Biochemical testing involved estimation of the postprandial capillary blood glucose level on campus. Additionally, systolic blood pressure and diastolic blood pressure were measured.

The cut-off point for waist circumference was less than 88 cm which was considered normal but 88 or more was considered high waist circumference. For postprandial blood sugar if the value is 140 mg/dL or more, it was considered as prediabetic and if 200 mg/dL or more, it was considered diabetic. As regards systolic blood pressure, 120mm/mg or more was considered elevated. For diastolic blood pressure, 80 mm/dL or more was considered elevated.

The physical activity score was considered high, if there was vigorous-intensity activity for at least 60 minutes on at least three days per week. The score was moderate, if there are three or more days of vigorous activity of at least 30 minutes per day or five or more days of moderate-intensity activity or walking of at least 30 minutes per day.

Statistical analysis 

Data were collected and grouped by using Microsoft Excel. Statistical analyses were performed using IBM SPSS Statistics for Windows, Version 22 (Released 2013; IBM Corp., Armonk, New York, United States). The normality was tested with the Shapiro-Wilk test. Normally distributed data are presented as mean ± standard deviation (SD). For frequency calculations, percentages were used. Correlation tests (Pearson’s χ^2^ test or Fisher’s exact test) were used for analyzing qualitative variables. The level of significance adopted was p < 0.05.

Ethical consideration 

Approval for this study were obtained from the Ethical Committee of Ibn Sina National College for Medical Studies (IRRB Ref No.: IRRB-02-28022022). All information obtained was kept strictly confidential. The data collection sheet included consent for participation.

## Results

Sociodemographic data among female medical students at a private college in Saudi Arabia are illustrated in Table 1. A total of 272 female medical students aged between 18 and 29 participated in the study. Data were normally distributed. The mean age was 21.51 years with 2.18 standard deviation. Skewness and Kurtosis were identified as acceptable which were .587 skewness and 1.38 kurtosis. 

**Table 1:** Sociodemographic data among female medical students at a private college in Saudi Arabia (n=272).

Sociodemographic data	Frequency	Percentage
Age categories in years		
< 20	49	18.0
20 – 22	139	51.1
23 – 25	73	26.8
25 – 28	10	3.7
> 28	1	0.4
Level of education		
First	45	16.5
Second	63	23.2
Third	49	18.0
Fourth	65	23.9
Fifth	27	9.9
Sixth	23	8.5
Smoking status		
Never	237	87.1
Former	18	6.6
Current	17	6.3

Table 1 presents the different age categories of participants, showing that just more than half of the female students were in the age group 20 to 22 years old. On the other hand, only a few (0.4%) were above 28 years old. Students who participated in this study were from all levels but mainly from the second year (23.2%) and fourth year (23.9%). As regards smoking, a tiny fraction (6.3%) of the females were smokers.

The health status of the participants is illustrated in Table 2.

**Table 2:** Health status data among female medical students at a private college in Saudi Arabia (n=272). ^⋆^ASCVD: Atherosclerotic cardiovascular disease

History	Frequency		Percentage
Has dyslipidemia			
Yes		7	2.6
No		265	97.4
Has diabetes			
Yes		1	0.4
No		271	99.6
Has hypertension			
Yes		2	0.7
No		270	99.3
Has any hormonal disorders			
Yes		13	4.8
No		259	95.2
Has (ASCVD)^⋆^			
Yes		2	0.7
No		270	99.3
On corticosteroids			
Yes		11	4.0
No		261	96.0
Has a family history of dyslipidemia			
Yes		88	32.4
No		184	67.6
Has a family history of diabetes			
Yes		141	51.8
No		131	48.2
Has a family history of hypertension			
Yes		104	38.2
No		168	61.8
Has a family history of (ASCVD)^⋆^			
Yes		51	18.8
No		221	81.3

The vast majority did not have dyslipidemia (97.4%), diabetes mellitus (99.6%), hypertension (99.3%), hormonal disorders (95.2%), or cardiovascular diseases (99.3%). Additionally, a minority (4.0%) of the sample were on corticosteroids. However, 32.4% of the participants have a positive family history of dyslipidemia. Slightly above half (51.8%) of the sample have a family history of diabetes mellitus. More than one-third (38.2%) of the sample have a family history of hypertension. Less than one-fifth (18.8%) have a family history of cardiovascular diseases.

Table 3 shows the prevalence of risk factors known to be associated with IR among female medical students at a private college in Saudi Arabia.

**Table 3:** Prevalence of risk factors known to be associated with insulin resistance among female medical students at a private college in Saudi Arabia, 2022 (n=272).

Risk factors		Frequency	Percentage
Body mass index	Normal BMI	189	69.5
	Overweight or obese	83	30.5
Waist circumference	Normal waist	209	76.8
	High waist	63	23.2
Blood sugar	Normal	260	95.6
	Prediabetic or diabetic	12	4.4
Systolic blood pressure	Normal systole	169	62.1
	Elevated or high systole	103	37.9
Diastolic blood pressure	Normal diastole	158	58.1
	Elevated or high diastole	114	41.9
Stress score	Least likely to suffer from stress	96	35.3
	Prone to stress	176	64.7
Night sleep	Good sleep	74	27.2
	Poor sleep	198	72.8
Diet score	Healthy diet	58	21.3
	Unhealthy diet	214	78.7
Physical activity	Physically active	180	66.2
	Physically inactive	92	33.8

Less than one-third (30.5%) of the female participants were overweight or obese. Similarly, less than one-fourth (23.2%) had high waist circumferences. As regards blood sugar levels, a minority (4.4%) were found to be pre-diabetic or diabetic. However, more than one-third (37.9%) had elevated or high systolic blood pressure. Moreover, 41.9% had elevated or high diastolic blood pressure. Of the total medical students who participated in this study, most of the participants had three parameters considered unhealthy lifestyle which was prone to stress (64.7%), unhealthy diet (78.7%), and poor night sleep (72.8%). On the contrary, almost two-thirds (66.2%) of the participants were physically active.

The association of lifestyle risk factors with waist circumference as an indicator of IR using Chi-square is illustrated in Table 4.

**Table 4:** Association of lifestyle risk factors with waist circumference as an indicator of insulin resistance among female medical students in a private college in Saudi Arabia (n=272).

Variables		Waist circumference n= 272				Chi	P-value
		Normal waist n=209		High waist n=63			
		Frequency	percentage	frequency	percentage		
Physical activity	Physically active	156	74.6%	24	38.1%	28.9	0.000*
	Physically inactive	53	25.4%	39	61.9%		
	Total	209	100.0%	63	100.0%		
Stress	Least likely to suffer from stress	82	39.2%	14	22.2%	6.2	0.013*
	Prone to stress	127	60.8%	49	77.8%		
	Total	209	100.0%	63	100.0%		
Night sleep	Good sleep	54	25.8%	20	31.7%	0.85	0.35
	Poor sleep	155	74.2%	43	68.3%		
	Total	209	100.0%	63	100.0%		
Diet	Healthy diet	47	22.5%	11	17.5%	0.73	0.393
	Unhealthy diet	162	77.5%	52	82.5%		
	Total	209	100.0%	63	100.0%		

Less than two-thirds (61.9%) of female medical students with high waist circumferences participated in this study were physically inactive compared to those with normal waist circumference (p= .000) which is highly statistically significant. Additionally, the majority (77.8%) of those with high waist circumference were more prone to stress and it is statically significant when compared to those with normal waist circumference (p = .013). Moreover, more than two-thirds (68.3%) of those with high waist circumference showed poor sleep patterns at night which is not statistically significant (p = .355. Finally, an unhealthy diet was more associated with most of those with high waist circumference (82.5%); however, it is not statistically significant (p = .393).

**Figure 1:**
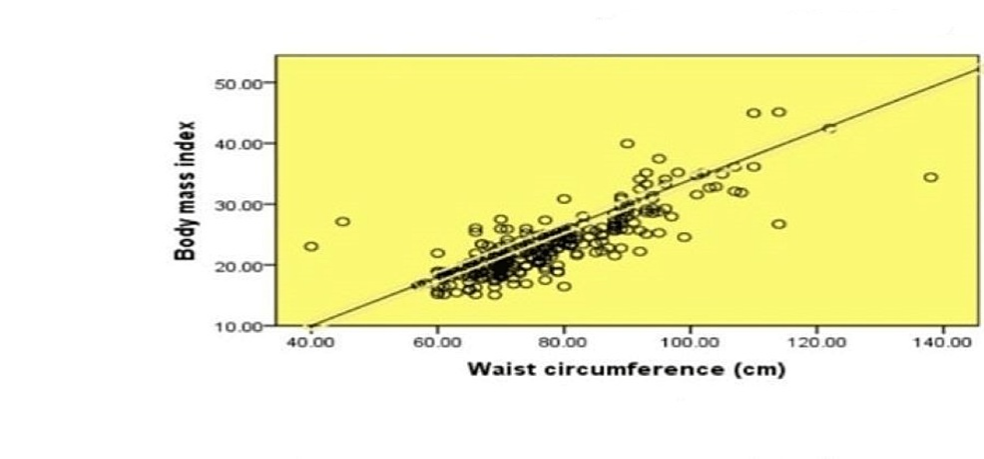
Correlation between waist circumference and body mass index. A scatter plot showing the correlation between waist circumference as an indicator of insulin resistance and body mass index among female medical students at a private college in Saudi Arabia. Based on the correlation p = 000^⋆^ ^⋆^means significant correlation

**Figure 2:**
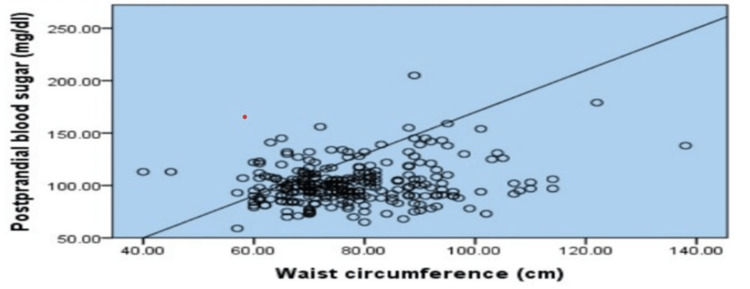
Waist circumference and postprandial blood sugar level A scatter plot showing the correlation between waist circumference as an indicator of insulin resistance and postprandial blood sugar level among female medical students of a private college in Saudi Arabia. Based on the correlation p = 000^⋆^ ^⋆^means significant correlation

**Figure 3:**
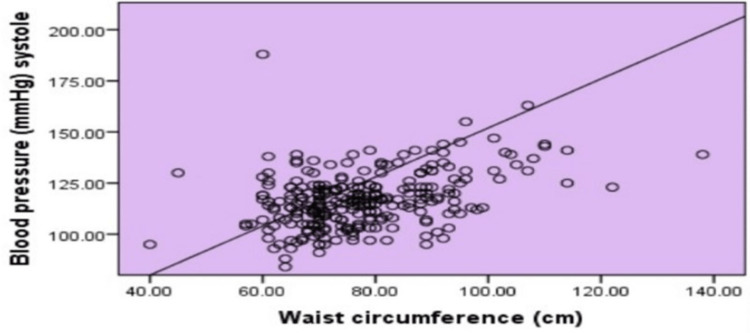
Correlation of waist circumference and systolic blood pressure A scatter plot showing the correlation between waist circumference as an indicator of insulin resistance and systolic blood pressure among female medical students at a private college in Saudi Arabia. Based on the correlation p = 000^⋆^ ⋆means significant correlation

**Figure 4:**
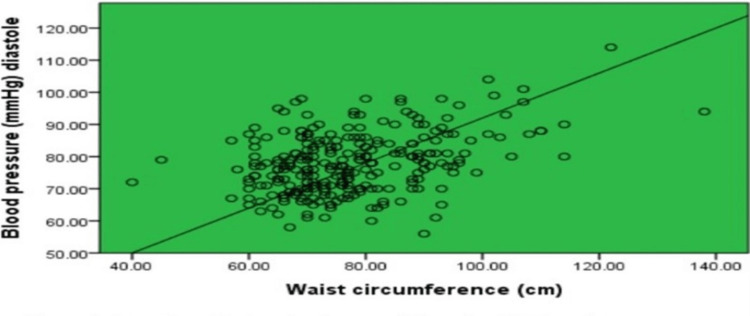
Correlation of waist circumference and diastolic blood pressure A scatter plot showing the correlation between waist circumference as an indicator of insulin resistance and diastolic blood pressure among female medical students at a private college in Saudi Arabia. Based on the correlation p = 000^⋆^ ⋆means significant correlation.

Scatters plots in Figures 1-4 show the correlation of waist circumference (cm) as an indicator of IR with BMI, postprandial blood sugar (mg/dl), systolic blood pressure (mmHg), and diastolic blood pressure (mmHg).

Overall, it was clear that all figures described a positive statistically significant relationship between body mass index (r = .815, p = .000), postprandial blood sugar (r = .222, p = .000), systolic blood pressure (r = .389, p = .000), and diastolic blood pressure (r = .378, p = .000).

## Discussion

A cross-sectional approach was carried out in which selective structural analysis has been adopted to expand our understanding of IR causes. The pathology of IR is influenced by the variability of genetic and environmental conditions [2]. However, the primary focus was to highlight modifiable risk factors which are seven factors that emerged in the present analysis including BMI, waist circumference, blood pressure, stress, night sleep, diet, and physical activity.

Considering the educational level of the female medical students, the majority were fourth-year and second-year students, whereas third, fifth-, and sixth-year students were the least recorded. The reason for this variation seems likely that these results are in fact due to the different type of commitment each level demands. The fourth-year student participants were enrolled in a research course that year which makes their commitment the highest. For the second-year students, they were engaged in community service on campus, so they were able to participate in this study outside their classrooms. According to the National Institute of Diabetes and Digestive and Kidney Diseases, obesity, especially too much fat in the abdomen and around the organs (central obesity), is one of the main causes of IR [17]. The current study found a high prevalence (30.5%) of obese and overweight medical female students. This finding strongly supports a recent study conducted among 202 Omani male and female students which found that 28% were overweight or obese [18]. In addition, the results of the current study found that 23.2% of female medical students had a high waist circumference which is considered the main indicator of IR. These findings agreed with the results revealed by research [19-21].

The study demonstrates that about one-third (37.9%) of students had elevated or high systolic blood pressure, and 41.9% had elevated or high diastolic blood pressure. This very high level can be explained by the fact that the cut-off points for both systolic and diastolic blood pressure were as follows: The new blood pressure categories: the elevated blood pressure is 120 mmHg for systole and 80 mmHg for diastole which are indicated in the hypertension guidelines of 2017 [22]. Most of the participants had an unhealthy lifestyle which was indicated by the level of stress, enough night sleep, healthy diet, and physical activity level. 

The current study revealed that around two-thirds (64.7%) of the participants were prone to stress. Several researchers have shown a prevalence of stress among medical students in Saudi Arabia (28.9%), Egypt (30.9%), and Malaysia (41.9%). In another literature study, they attributed this stress to the fact that medical students are overloaded with a tremendous amount of information as well as they have a limited amount of time to memorize all the information studied. The overload of information creates a feeling of disappointment because of the inability to handle all the information at once and succeed during the examination period [23]. Additionally, the majority (72.8%) of the participants in the current research had poor night sleep as it was reported by several studies among medical students [24,25].

An unhealthy diet was common among the majority (78.7%) of the female medical students. These results corroborate the findings of those mentioned in a previous study that most of the medical students of King Abdelaziz University had unhealthy dietary habits [26]. The unhealthy dietary habits were irregular meals, inadequate consumption of fruits and vegetables, and eating too many fried and fast foods. In Saudi Arabia, eating patterns have altered considerably during the past few decades. These marked lifestyle changes have affected different age groups, especially children and youth [27].

As regards physical activity, around two-thirds (66.2%) of the female medical students were physically inactive. These results match those mentioned in earlier studies at King Abdelaziz University, Jeddah, Saudi Arabia. The study on the risk factors of cardiovascular disease revealed that physical inactivity was common among 57.9% of medical students [28]. A cross-sectional study on 319 Egyptian and 297 Saudi medical students showed that 41.1% of Saudi medical students were physically inactive compared to 15.4% of Egyptian medical students [29]. These results of low physical activity level among medical students may be due to busy academic schedule, time limitation, having other priorities, unsuitable weather, fear of injuries, and previous bad experience with sports.

The main objective of this study was to find the predictability of the development of IR based on the risk factors among female medical students. The relation of waist circumference as an indicator of IR and the various lifestyle risk factors was studied. Less than two-thirds of female medical students with high waist circumference who participated in this study were physically inactive compared to those with normal waist circumference which is statistically significant. The results of the current study were matched with a cross-sectional study in the Indonesian middle-aged adult rural population which demonstrated that there was a relationship between physical activity and the incidence of central obesity in middle-aged people in rural populations [30]. Additionally, the majority of those with high waist circumference were more prone to stress and it is statically significant when compared to those with normal waist circumference [31]. In a study by Kyrou and Tsigos, they found that greater exposure to life stress or psychological vulnerability to stress enhanced cortisol reactivity. In turn, the cortisol exposure may have led to accumulation of greater abdominal fat. Cortisol affects fat distribution by causing fat to be stored centrally around the organs. Moreover, chronic stress is associated with enhanced vulnerability to diet-related metabolic risk like abdominal adiposity, IR, and oxidative stress [32]. Recent data indicate that chronic stress, associated with mild hypercortisolemia and prolonged nervous activation, favors accumulation of visceral fat and contributes to the clinical presentation of visceral obesity, type 2 diabetes, and related cardiometabolic complications [33].

On the other hand, poor night sleep and unhealthy diet were not statistically significant between those with high waist circumference and those with low waist circumference. Although the prevalence of both variables was high among female medical students and not statically significant between both groups of waist circumference, it is still an alarm for them to develop IR in the near future. That is, waist circumference was a significant and independent predictor of lifestyle determinants in the present study.

As regards the correlation between waist circumference and BMI, a strong positive correlation was revealed which was statistically significant where r = .815. These results can be explained by the fact that most of the individuals who are obese have a high waist circumference [34]. Central obesity has been identified as an important determinant of type 2 diabetes risk [35]. The correlations estimated in this study confirmed that waist circumference was positively and significantly associated with diabetes risk with a relatively weak correlation (r = .222). These findings were emphasized by a study in Egypt, which revealed that, after adjustment of other confounding factors by logistic regression, waist circumference emerged as a more powerful predictor of diabetes risk [34]. An association between hypertension and central obesity has also been reported. Such an association was evident in the present study based on both waist circumference and both systolic and diastolic blood pressure levels. That is, waist circumference was a significant and independent predictor of both diabetes mellitus and hypertension in the present study.

Study limitation

Limitations of this study include that it was conducted among young female medical students. The sample was not representative of the population due to differences in risk factors among the cohort as compared to the general Saudi population, as well as the narrow age range and lack of male participants. The study was therefore limited by selection bias. Different examiners were also used for taking anthropometric measurements, which may give rise to inter-examiner variability. Lastly, questionnaires for physical activity, stress, night sleep, and diet were based on self-reported data. Due to the subjectivity of such data, participants may under-report or over-report certain factors.

## Conclusions

This study's target was to correlate predictability of developing IR and risk factors encountered by female medical students. A series of unhealthy lifestyle habits is contributable to developing obesity and therefore IR among those students. The overload of medical school leads to stress in which the resultant is significant physical inactivity and unhealthy eating habits, which is expressed by developing IR. High waist circumference is a significant indicator for detecting IR.

Recommendation

Further screening and awareness camping are recommended for modification of lifestyle habits toward IR and to focus on primordial prevention techniques, also, to establish a more simplified regimen for increasing insulin sensitivity, and to be up to date with arising new risk factors.
